# Terrestrial laser scanning for 3D mapping of an alpine ice cave

**DOI:** 10.1111/phor.12437

**Published:** 2022-12-29

**Authors:** Jan Pfeiffer, Martin Rutzinger, Christoph Spötl

**Affiliations:** ^1^ Department of Geography University of Innsbruck Innsbruck Austria; ^2^ Institute for Interdisciplinary Mountain Research Austrian Academy of Sciences Innsbruck Austria; ^3^ Institute of Geology University of Innsbruck Innsbruck Austria

**Keywords:** change detection, ice cave, principal component analysis (PCA), principal components, terrestrial laser scanning (TLS)

## Abstract

Perennial ice deposits in caves are an underexplored component of the cryosphere preserving a largely untapped archive of long‐term changes in landscape and climate whose existence is threatened by climate change. This study demonstrates how terrestrial laser scanning (TLS) can be used to fully and accurately (registration accuracy < 1 cm standard deviation of point differences) assess the geometry of an ice‐bearing cave in the Eastern Alps (Tyrol, Austria). Three TLS campaigns and 255 scan positions were used to acquire point clouds with a high sampling density (2 cm average point spacing) in order to minimise shading effects and to assure a precise and highly resolved 3D documentation of the cave. A semi‐automated registration and point cloud‐processing approach adapted to the site‐specific demands ensured a complete and error‐minimised assessment of the cave's geometry serving as a solid basis for future quantifications of snow and ice content dynamics. Dominant cave surface structures were investigated by performing a multiscale principal component analysis (PCA) to identify a detailed and computationally efficient basis for future airflow modelling tasks.

## Graphical abstract

Perennial ice deposits in caves are an underexplored component of the cryosphere preserving a largely untapped archive of long‐term changes in landscape and climate whose existence is threatened by climate change. This paper demonstrates how TLS can be used to fully and accurately assess the geometry and snow, respectively ice, content dynamics of an ice‐bearing cave. The semi‐automated registration and point cloud‐processing approach proved successful and represents a solid computational efficient basis for future airflow modelling tasks.

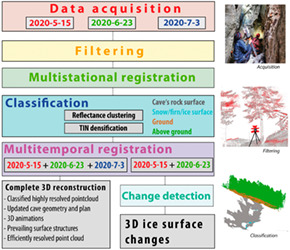

## INTRODUCTION

Mapping of caves is conventionally done by a combination of distance meter, compass and clinometer (Heeb, 2014; Trimmis, 2018), and in special cases total stations. These measurements are carried out at single points or along profiles entailing limitations of spatial coverage and preventing the acquisition of a spatially highly resolved and complete map of a cave's geometry. The increasing availability of remote sensing techniques and access to computational resources has resulted in a wide use of remotely sensed data in geosciences. Sensors, acquiring information of the close‐range environment, are well suited for a detailed mapping of cave geometries. In recent years, photogrammetric and laser scanning technologies were also applied in speleological research (Fabbri et al., 2017; Idrees & Pradhan, 2016). Data acquisition and 3D data processing (e.g., registration) became more efficient and partially automated. Furthermore, sensors and other used equipment became smaller and more compact permitting efficient mapping of large and complex structures, also in topographically and logistically challenging environments.

Mapping a cave's 3D geometry is performed for a number of purposes. The 3D documentation and visualisation of cultural heritage sites were among the first tasks that involved a combined laser scanning and high‐resolution photography approach to create photo‐realistic models of highly protected caves (Caprioli et al., 2003; El‐Hakim et al., 2004). The field of archaeological documentation often demands both terrestrial laser scanning (TLS) in order to derive accurate and high‐resolution 3D models combined with close‐range photogrammetry to additionally record their photo‐realistic characteristics (Lerma et al., 2010). Where photogrammetric methods for surface reconstruction rely on an external light source, the advantage of laser scanning as an active remote sensing technique is that data can be acquired in complete darkness permitting a convenient data acquisition in caves lacking artificial lighting (Fryer et al., 2005). Besides documentation of archaeological sites, other applications are more related to investigations of 3D structures which can accurately be assessed by laser scanning sensors. Among them, TLS of cave morphologies offers data of unprecedented precision and resolution for speleogenetic studies (Gallay et al., 2015; Idrees & Pradhan, 2016, 2017). Different approaches have been developed and applied to analyse 3D geomorphological cave structures in order to advance the understanding of their formation and underlying geological processes. De Waele et al. (2018) focused on the reconstruction and analysis of the evolutionary stages of paragenetic morphologies such as ceiling channels and pendants. Hämmerle et al. (2014) tested and investigated the performance of low‐cost laser scanning devices for mapping cave features. Silvestre et al. (2015) and Gallay et al. (2015) presented studies demonstrating the limitations, challenges and potentials of TLS in caves. Based on this, Silvestre et al. (2015) included the presentation of two algorithms for determining dimensions of stalactites on cave ceilings.

Perennial ice deposits are locally present in alpine caves and caves located at high latitudes. These deposits originate from snow falling into these caves and/or by freezing of drip water and represent a largely untapped and promising archive of past environmental and climate change (Luetscher, 2013). In order to assess the fate of these underground glaciers in a warmer world it is important to quantify seasonal ice mass‐balance changes (Securo et al., 2022). TLS offers a unique technique to monitor ice volume changes in caves in a matter of hours to days depending on the cave's accessibility and dimensions. In this context Buchroithner and Gaisecker (2020) presented an illustrative study on quantifying ice surface changes in Eisriesenwelt, a large ice cave in the Tennen Mountains (Salzburg, Austria), covering the period between 2017 and 2020. Šupinský et al. (2019) introduced additional improvements in detecting dynamics of cave floor ice by addressing registration issues with a selective cloud‐to‐cloud approach.

The number of studies available and demonstrating the applicability of close‐range remote sensing techniques for mapping ice‐bearing caves is very limited (e.g., Buchroithner & Gaisecker, 2020; Securo et al., 2022; Šupinský et al., 2019). At the same time, ice caves experience high melting rates threatening their existence under warming climate (Colucci & Guglielmin, 2019; Kern & Perşoiu, 2013). A detailed and three‐dimensional documentation of current ice‐fillings and ice dynamics is therefore key to preserve fundamental information of these vanishing paleo‐environmental archives and tourist attractions (Bella et al., 2020; Sancho et al., 2018; Securo et al., 2022).

Moreover, the study of air flow in caves using numerical models is of great potential for deriving a deeper understanding of the controls of cave ice dynamics. Besides atmospheric parameters, these models demand for a detailed and computationally efficient representation of the complex geometrical structures found in caves (Bertozzi et al., 2019; Jarosch & Obleitner, 2022). At the same time no study applying numerical airflow models in ice‐bearing caves has been parameterised by a detailed remotely sensed 3D model representing a cave's real architecture. Whereas Bertozzi et al. (2019) used conventional topographic surveys and a computer‐aided design (CAD) reconstruction to approximate a cave's 3D model, Jarosch and Obleitner (2022) focused on the physically based modelling of air circulation and thermal conditions on a highly simplified geometry of a sag‐type cave.

A multitude of ice caves are operated as show caves demonstrating their significance as tourist attractions (Kudla, 2020). 3D animations of ice caves offer the unique opportunity to make them accessible for virtual visitors (Buchroithner & Gaisecker, 2020). On‐site visitor fluxes at the same time demand for profound mitigation concepts ensuring a safe operation and protection against, for example, rock fall. In this context, existing and highly resolved 3D models can represent a powerful dataset supporting the mitigation of rock fall hazard. Instable rocks can be localised, block sizes can be measured, and probable failure mechanisms and zones of instability can be identified by the help of 3D data (Fanti et al., 2013; Idrees & Pradhan, 2018; Wichmann et al., 2019).

While the above‐mentioned benefits and opportunities of (multi‐temporal) 3D models of ice‐bearing caves are obvious, the acquisition of a ready‐to‐explore clean data set is not entirely trivial and comes with several challenges. Among them, omnipresent moist and cold conditions are known to cause a multitude of outliers demanding for simple and efficient removal strategies. The often‐complex geometry and accessibility challenge a complete acquisition of the cave's surface. The plurality of scan positions and individual point clouds provoke a carefully balanced acquisition procedure and registration approach. Time‐consuming manual assistance (e.g., setting of scan properties) are required by the operator before each scan acquisition. Post‐processing of point clouds acquired by hundreds of scan positions and their integration into a combined reference system requires time‐consuming operator interactions when working with state‐of‐the‐art non‐automated registration methods (e.g., registration by equivalent point‐picking, target‐based registration, registration of already coarsely aligned point clouds using the iterative closest point algorithm; Gallay et al., 2015; Lichti & Skaloud, 2009). Moreover, large‐scale surface changes caused by potential ice melting or accumulation processes intensify required registration work and demand for an application of more complex alignment methodologies (Wujanz et al., 2016).

Tackling these challenges, this study presents a highly automated and site‐adapted acquisition and processing workflow to create complete, outlier‐cleaned and co‐registered point clouds. The workflow was applied to TLS data of the Hundsalm ice cave (Tyrol, Austria) acquired during three campaigns in spring and early summer of 2020. By exploiting an inertial measurement unit (IMU)‐integrated acquisition mode, a time‐efficient data acquisition was ensured by simultaneously recording relevant parameters supporting the consecutive and automated registration of 255 scan positions. In‐depth data exploration facilitated the determination of appropriate filter thresholds identifying outliers of different origin. Separate treatment of points belonging to the cave's exterior and interior and the application of custom‐tailored classification approaches allowed us to group them into four different surface types (cave's rock surface, cave's snow/firn/ice surface, exterior ground surface and exterior above ground surface). The snow/firn/ice covered and highly dynamic parts of the cave could be left out in order to facilitate transformation parameters towards an accurate multi‐temporal alignment of the three different campaigns that contain different levels of snow/firn/ice. Finally, a complete 3D reconstruction of the cave could be retrieved permitting the quantification of surface changes on the snow‐ and ice‐covered parts between the different acquisition campaigns. Additionally, the reconstruction was used to analyse and identify a computationally efficient spatial resolution that still captures the cave's structural characteristics, but is also applicable to air circulation models for studying energy fluxes and related ice‐dynamics using a custom‐built approach of multi‐scale principal component analyses (PCAs).

In summary, the objectives of this study include (1) a complete mapping of the 3D geometry of the cave, (2) a quantitative description of the cave's geometry, (3) a first assessment of short‐term ice and snow dynamics, and (4) an analysis of prevailing cave surface structures for the identification of a suitable spatial resolution to facilitate the application of numerical airflow models.

## STUDY SITE

**FIGURE 1:**
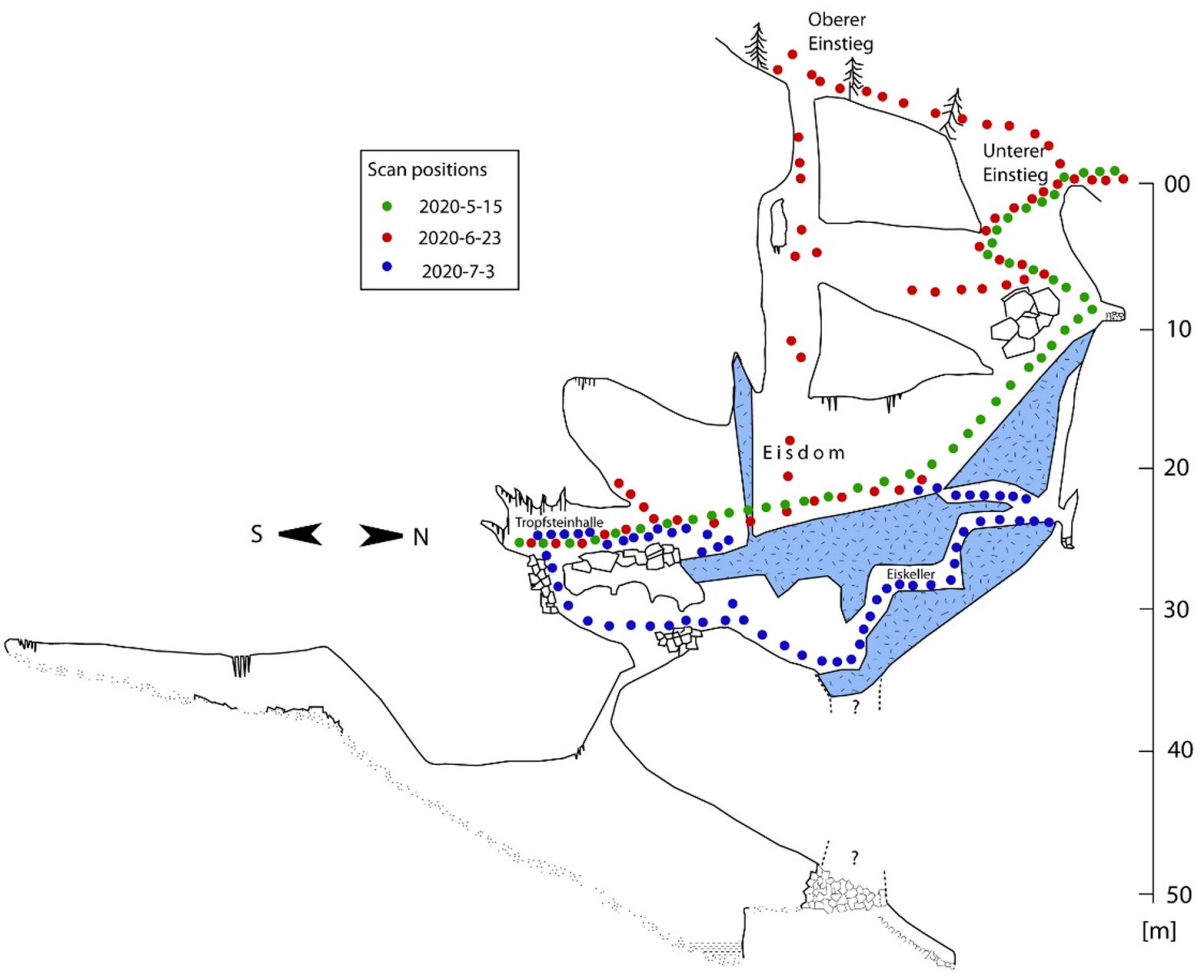
Acquisition set‐up sketched on a vertically projected plan of Hundsalm ice cave with the approximate scan positions for the three acquisition campaigns.

Hundsalm ice cave is a subvertical cave located at 1520 m asl in the Northern Calcareous Alps north of the city of Wörgl in Tyrol (Austria). First explored in 1921, the cave was opened for public visitors in 1967. Two adjacent shaft‐like connections to the surface (“Oberer Einstieg” and “Unterer Einstieg”) (Figure 1) trap cold and dense winter air because the cave lacks a lower entrance. Snow falling and sliding into these shafts as well as ice formed by freezing of drip water partly survive the summer period. Most of the firn and ice body formed during the second half of the Little Ice Age (approximately 15th–17th centuries; Spötl et al., 2013). Recent decades have shown a steady decrease in ice height and volume (Wind et al., 2022) similar to other small sag‐type ice caves in the Alps (Spötl et al., 2018) and elsewhere (Kern & Perşoiu, 2013).

A tourist walkway consisting of metal staircases, bridges, roofs and handrails allow easy access to the upper part of the cave by entering via the Unterer Einstieg. The floor of this upper part is largely covered by snow, firn and ice containing some rock fragments. The ice thickness decreases with increasing distance from the two shafts. The southern part of the cave contains some seasonal ice in spring. The deeper level of the cave is completely ice‐free and the narrow connection (which was artificially opened in 1986) is closed by a door to prevent air exchange with the upper, colder cave part.

## MATERIALS AND METHODS

### Data acquisition

The upper part of Hundsalm ice cave, which is operated as a show cave, was mapped by means of TLS. Three acquisition campaigns were performed to ensure a complete acquisition of the topographically challenging surface structures. Acquisitions were carried out on 15 May, 23 June and 3 July 2020 using a Riegl VZ‐2000i TLS operating with a near‐infrared wavelength (*λ* = 1550 nm). A laser pulse repetition rate of 1.2 MHz was selected for an efficient and fast acquisition (approximately 30 s per scan position) of the cave structures allocated in the close‐range environment. Scan positions were arranged along trajectories in the cave including the outside environment above the cave. The scanner‐integrated IMU combined with the one touch acquisition mode allowed a fast change between the automatically created scan positions demanding only one action (“touch”) by the operator per scan position. Reconstructed trajectories supported the later registration of consecutive scans (RIEGL Laser Measurement Systems GmbH, 2022a). The distance between the individual scan positions was preselected to be within a range of 1–3 m according to the local conditions for positioning a tripod and with the aim to capture the entire cave geometry by minimising shading effects. The approximate course of individual trajectories within the cave is sketched in Figure 1. A lightweight and quickly adjustable tripod was used in order to facilitate the transport of the scanner between the positions and to support a straightforward and flexible acquisition even at rough, narrow and steep parts of the cave. Greater distances had to be accepted for the trajectory in the subvertical upper entrance (Oberer Einstieg). Finding suitable locations for deploying the tripod was challenging in this part of the cave where the acquisition was performed along a safety rope. Major differences in snow and ice content were visually observed between 15 May and 23 June 2020. Therefore, potential surface modifications at the snow/ice/firn surface had to be considered in the succeeding registration of multi‐temporal point clouds and data analysis.

The first acquisition on 15 May 2020 explored the feasibility and detection of potential challenges for processing TLS data acquired in this cave. The trajectory started in the “Tropfsteinhalle” at the southern end of the cave (Figure 1), passed the big hall of the “Eisdom” and followed the main entrance (Unterer Einstieg) to the surface. The second campaign (23 June 2020) aimed at acquiring gapless data along the vertical shaft‐like upper entrance (Oberer Einstieg) and its upper connection to the lower main entrance (Unterer Einstieg). In order to facilitate future alignment of these data to those acquired during the first campaign, overlapping areas were included. The third campaign carried out on 3 July 2020 focused on the deepest parts of the cave. Here, the trajectory followed the narrow and tunnel‐like visitors' path from the Tropfsteinhalle through a passageway in snow and ice in the “Eiskeller”.

### TLS data processing

**FIGURE 2:**
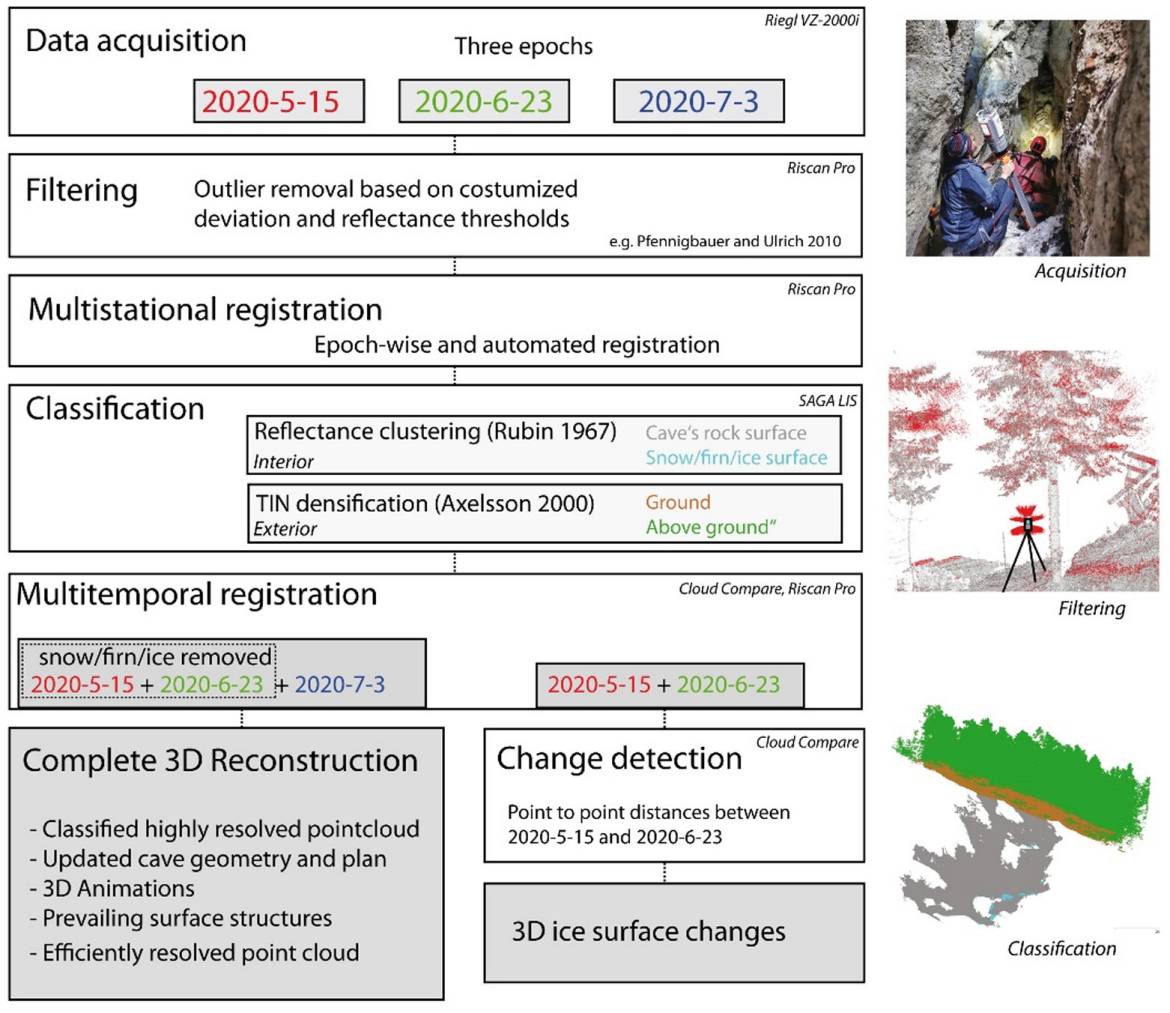
Data acquisition and processing workflow including filtering, registration, 3D reconstruction and change detection analysis.

A site‐ and target‐oriented workflow for processing acquired point clouds was deployed and is summarised in Figure 2. Observed outliers were eliminated by filtering. Point clouds from all three campaigns but containing different scenes of the cave were epoch‐wise registered onto each other to create a 3D reconstruction of the cave's inner surface. Supported by a site‐adapted classification approach, multi‐temporal registration was carried out in order to map changes in ice, snow and firn extent. This product was used to update the cave's projected vertical map, to estimate the cavity's volume, classify points regarding their surface representation, to create 3D animations of the data and to investigate how different spatial resolutions affect the representation of the caves surface structure.

### Outlier filtering and vegetation removal

Low temperatures and high humidity values in alpine caves often cause artefacts such as accumulated erroneous or phantom points (Charlton et al., 2009; Hasirlioglu et al., 2017). These points are frequently observable close the scanner's location and can be traced back to unusable reflections caused by humid air. They are characterised by low reflectance and high deviation. The reflectance is a scan‐range corrected attribute and therefore a suitable measure of backscattered intensity that allows comparisons between point clouds. The pulse deviation, as a measure of the points' reliability, indicates the influence of optical, electrical and digitisation noise (Pfennigbauer & Ullrich, 2010). In order to eliminate erroneous points, the scan distance was kept at a minimum and thresholds for deviation and reflectance were carefully selected based on the local and dataset depended requirements. Strongly differing reflectance values of backscattered signals between snow/firn/ice and rock surfaces were used to detect and (temporarily) eliminate snow/firn/ice surfaces that are assumed to change over time and therefore would disturb the registration process. Application of a clustering approach proposed by Rubin (1967) and implemented in SAGA geographical information system (GIS) classified underground (cave) points into snow/firn/ice and rock surface using the reflectance attribute (Wichmann, 2015). Cluster results were cleaned using a majority filter specified with a radius defining the local neighbourhood of 0.2 m. The points outside and above the cave were classified as vegetation (above ground) and non‐vegetation (ground) points by applying the progressive TIN densification method after Axelsson (2000). Finally, a classified and outlier‐filtered baseline of 255‐point clouds was created in order to enable a multi‐stational and multi‐temporal registration.

### Registration approach

To allow an efficient alignment of 255 scan positions a registration approach satisfying the site‐, data‐ and acquisition‐specific demands was deployed. To comply with the predefined aims of deriving a complete 3D reconstruction of the cave and to permit snow‐ and ice‐surface change determination between two campaigns, two diverging registration approaches were applied. Pre‐processed (filtered and classified) point clouds from 15 May, 23 June and 3 July 2020 were registered onto each other in order to derive a complete 3D reconstruction. Starting with the first scan position of 23 June all the other positions were successively registered thereon using the automatic registration approach. Automatic registration was performed within the Riegl RiSCAN PRO software (RIEGL Laser Measurement Systems GmbH, 2022b). This approach uses additionally recorded data from the scanner's internal global navigation satellite system (GNSS), accelerometers, magnetic digital compass, IMU and barometer to coarsely position the to‐be‐transformed point cloud. Based on voxel matching at overlapping areas the consecutive scan position is finely aligned. Manual assistance was necessary in case of an insufficient coarse alignment. In a second step, the individual scan positions from the outlier‐and snow/firn/ice surface filtered campaigns of 15 May and 3 July 2020 were registered on top of the data of 23 June 2020. In order to allow subsequent analysis of changes in snow and ice content between 15 May and 23 June, point clouds of these two campaigns were individually registered in a separate registration project. Based on assumed stable parts within these two dates, transformation parameters were derived and applied by using the Iterative Closest Point (ICP) algorithm (Besl & McKay, 1992).

### Gridding and volume estimation

A point cloud representing the complete 3D reconstruction of the cave was aggregated to a raster projected along the east–west striking *x*‐axis. The resulting map showing the acquired data on a vertically projected plane enabled a good comparison with an existing cave map obtained using a conventional cave survey. Point cloud attributes such as number of points, average reflectance and the minimum and maximum *x*‐values were used to aggregate a respective raster in order to visualise the cave's extent and calculate raster differences to indicate the horizontal cave depth. This depth‐map was further used to approximate the cave volume by multiplying the cave‐depth raster values with respective cell sizes. The map showing the number of points per raster cell indicates the point density and the quality of coverage.

### 3D change detection

For the assessment of expected changes of the snow/firn/ice surface, these areas were initially omitted for the derivation of transformation parameters. Re‐adding parts of expected surface changes while transforming the whole point cloud finally qualified the application of geometric change detection methods. 3D changes in snow/firn/ice surface height between 15 May and 23 June 2020 were assessed by calculating distances between the closest point of one epoch to the next. Point‐to‐point distances were calculated between the uniformly resolved (2 cm spatial resolution) point clouds using CloudCompare (2022). The examination of calculated distances on stable rock surfaces was used to additionally discuss the multi‐temporal registration accuracy.

### Multiscale principal component analysis (PCA)

In order to identify a suitable point resolution that supports the efficient application of turbulent air flow models in Hundsalm ice cave, surface characteristics were systematically analysed and investigated. Two subsets covering 18 m of vertical and 16 m of horizontal extent were analysed. One of the subsets represents a hanging wall and the other a footwall of the fault‐oriented cave boundaries. An automated workflow was deployed to calculate eigenvalues and eigenvectors by applying a PCA (Grohmann et al., 2011; Park et al., 2012; Pauly et al., 2003; Pfeiffer et al., 2019). The PCA was performed within a constant neighbourhood of 10 points and for different point densities with a spatial resolution from 2 to 400 cm. The omnivariance, expressing the difference between the three eigenvalues (Pfeiffer et al., 2019), was used as an indicator of surface roughness. By comparing statistics (e.g., median and interquartile range—IQR) of resulting omnivariance values calculated for point clouds with different point spacings, structural differences and similarities between the subsets were assessed. Generally high omnivariance values at a certain point spacing were seen as indicator of a frequent occurrence of structures of the same size as the respective point spacing. Similar omnivarance statistics between different subsets at a certain spatial resolution indicate this resolution to be equally able to describe the same structures between different parts of the cave.

## RESULTS

### 3D cave geometry

**FIGURE 3:**
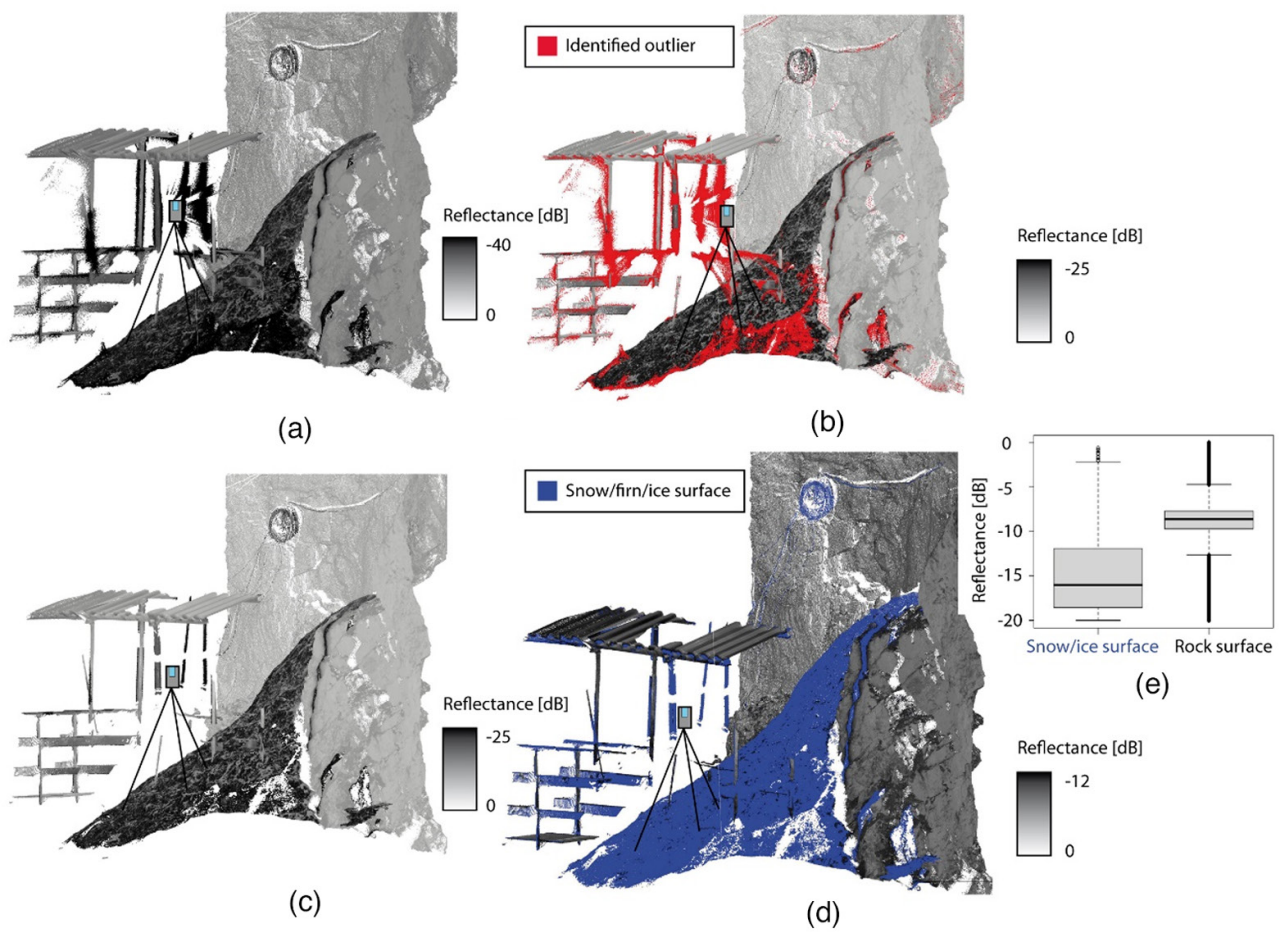
Exemplary point cloud showing results of the applied rule‐set‐based filtering approach: (a) reflectance‐coloured point cloud ranging from −40 to 0 dB; (b) outliers identified by using a threshold of < −25 dB leading to (c) an outlier‐removed clean point cloud; (d) snow and ice surface detection performed using a –12 dB reflectance threshold identified by previously performed analyses whose results are shown in (e).

Hundsalm ice cave was scanned by TLS with a mean distance of 1.6 m between individual scan positions. Point clouds from single scan positions include at least three points per 1 cm^2^ surface. The cold and moist conditions in this Alpine cave resulted in a high number of erroneous reflections which are unrelated to cave surface structures (Figure 3a). An explorative evaluation of the point cloud attributes has shown that a combined filtering approach utilising a rule set based on reflectance and deviation attributes facilitates the derivation of a clean error‐free point cloud. The effects and performance of the applied filtering approach are exemplarily shown in Figure 3b,c. A reflectance threshold of < 25 dB was identified to be well suited to eliminate these outliers. Further investigations on the reflectance differences of snow/firn/ice and rock surfaces have shown that a threshold of 12 dB allows a sufficient differentiation between these two surface properties (Figure 3d,e). This observation gives evidence for a promising application of the reflectance‐based clustering approach to differentiate between rock and snow/firn/ice surfaces.

**FIGURE 4:**
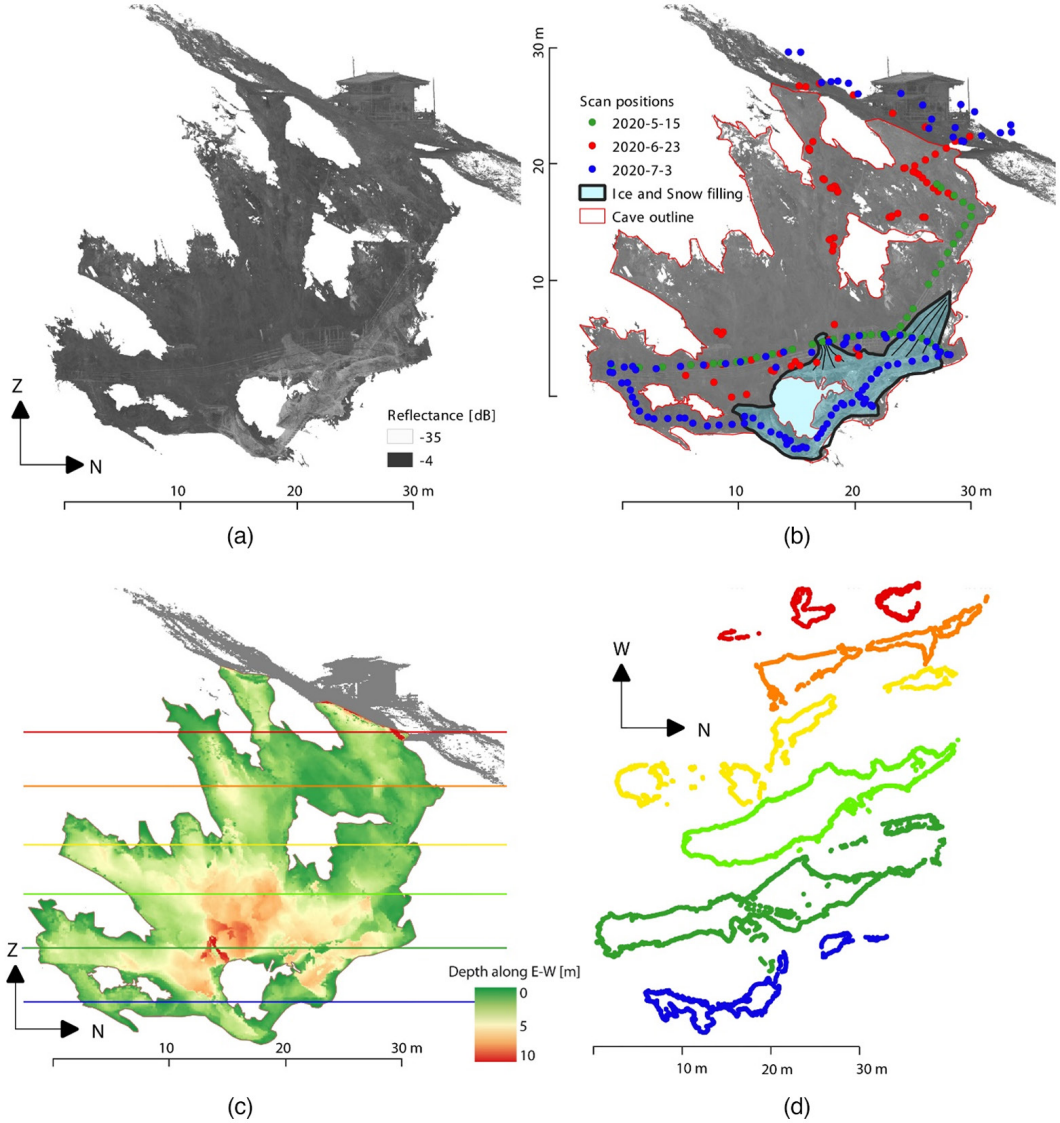
Map showing the aggregated point cloud of Hundsalm ice cave projected onto a vertical north–south striking plane: (a) mean reflectance values aggregated to a 10 cm raster allowing an indication of the snow‐ and ice‐filled part of the cave by its low reflectance values; (b) additionally inferred information indicating the cave outline and the assumed outline of the snow‐ and ice‐filling, where the snow cones beneath the two entrances are highlighted by thin black lines; final scan positions are located and colourised according to the respective acquisition campaign; (c) depth map showing the spatial distribution of maximum east–west extent; and (d) selected cave outlines extracted at different levels corresponding to equally coloured lines in (c).

Subsequent to outlier filtering and classification, snow‐ and ice‐containing points within the epochs 15 May and 23 June 2020 were removed and the complete cave geometry representing the snow and ice conditions of 3 July 2020 was derived. The overall registration accuracy is estimated to be < 1 cm (standard deviation of point differences). The complete and evenly thinned (2 cm point spacing) 3D reconstruction of the cave of 2 cm indicates a maximum depth of 33 m for the show cave part and a north–south extent of 33 m. Widths between the eastern and western cave's boundary are shown in a vertically projected depth map. They typically range from 1 to 7 m. A maximum width of 10 m along the east–west axis was obtained for the lower central part of the cave (Figure 4c). A selection of extracted cross‐sections along the vertical axis is shown in Figure 4d and indicates the high structural variability and complexity of the cave's outline.

The 3D data clearly illustrate that the cave follows a major fault with dip direction of 070 and a dip‐angle of 65. The cave volume is approximately 2500 m^3^. An updated map of the show cave along a north–south striking vertical transect is shown in Figure 4a. The reflectance‐coloured raster shown therein facilitates the localisation of the snow and ice fillings characterised by low values. Derived therefrom, the caves outline as well as the interpreted outline of the ice deposit with the distinct cone structures underneath the two shaft‐entrances were indicated and final scan positions for acquiring point clouds are included (Figure 4b).

### 3D changes of snow/firn/ice

**FIGURE 5:**
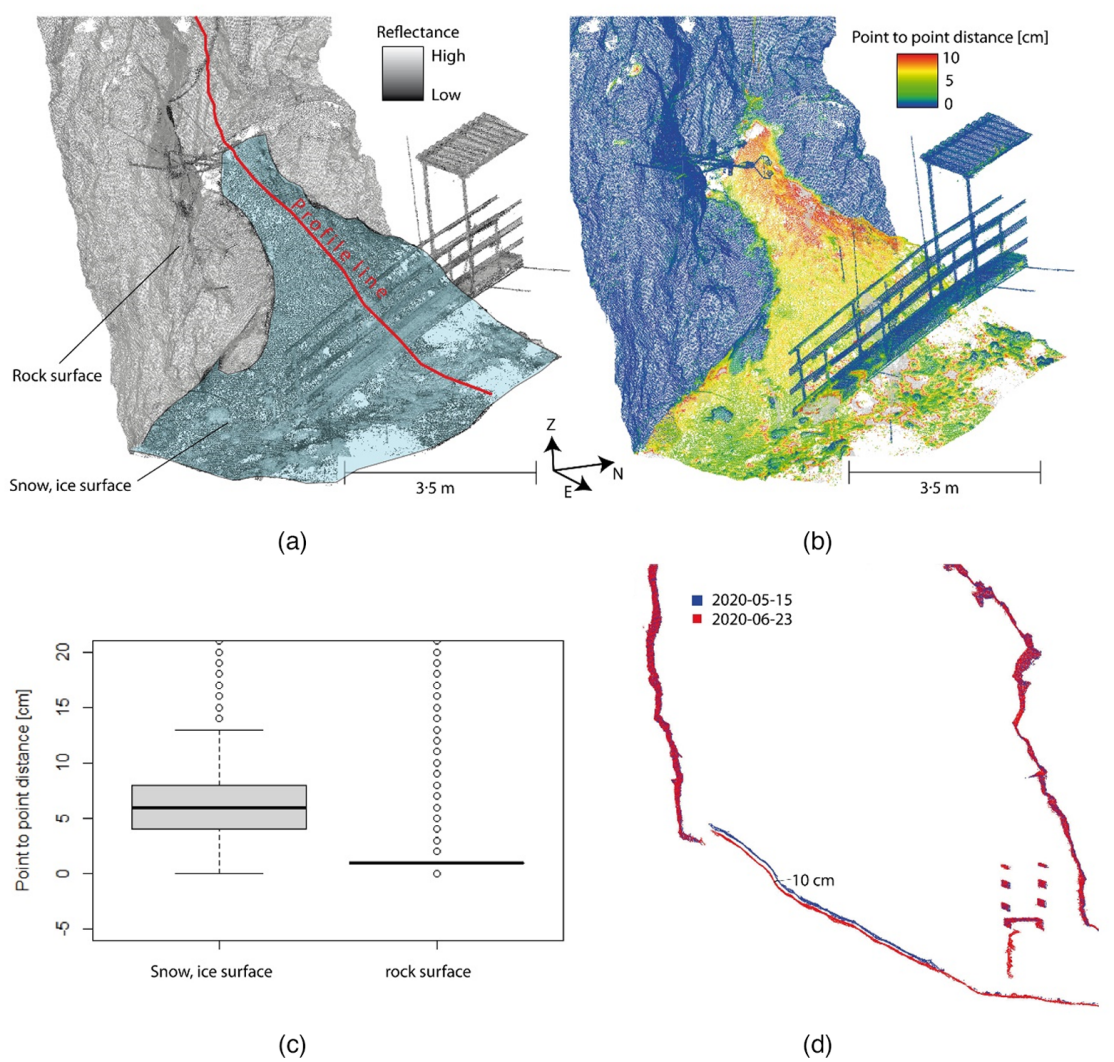
Results of quantified geometric changes between 15 May and 23 June 2020: (a) exemplary point cloud subset showing the snow cone below the upper cave entrance; (b) area‐wide assessed point‐to‐point distances whose numerical range is shown in (c) for snow/ice surfaces and rock surfaces; and (d) transect through the cave for 15 May and 23 June 2020.

Assessed surfaces changes are limited to the snow/firn‐ and ice‐covered parts and the spatially overlapping areas between the acquisitions of 15 May and 23 June 2020 (Figure 5a). The surface appearance of the ice‐filled part varies on a small scale and with time. In general, slightly debris‐covered parts of ice dominate the entrance‐distal regions. Areas in proximity to the shaft entrances are generally dominated by more clean snow or firn. The two cone‐shaped snow inlets close to the shaft‐entrances show maximum changes in surface elevation. Here, point‐to‐point distances of more than 10 cm indicate a distinct lowering of the snow/firn/ice surface. The further away from cone's apex the smaller the surface height difference (approximately 2 cm) (Figure 5b). Overall changes of approximately 6 cm indicate a lowering of the snow and ice surface between 15 May and 23 June 2020. The distances on the surrounding rock are approximating < 1 cm (Figure 5c,d). This comparison confirms the registration quality and indicates a detection limit in the 1 cm range.

### Surface structures

**FIGURE 6:**
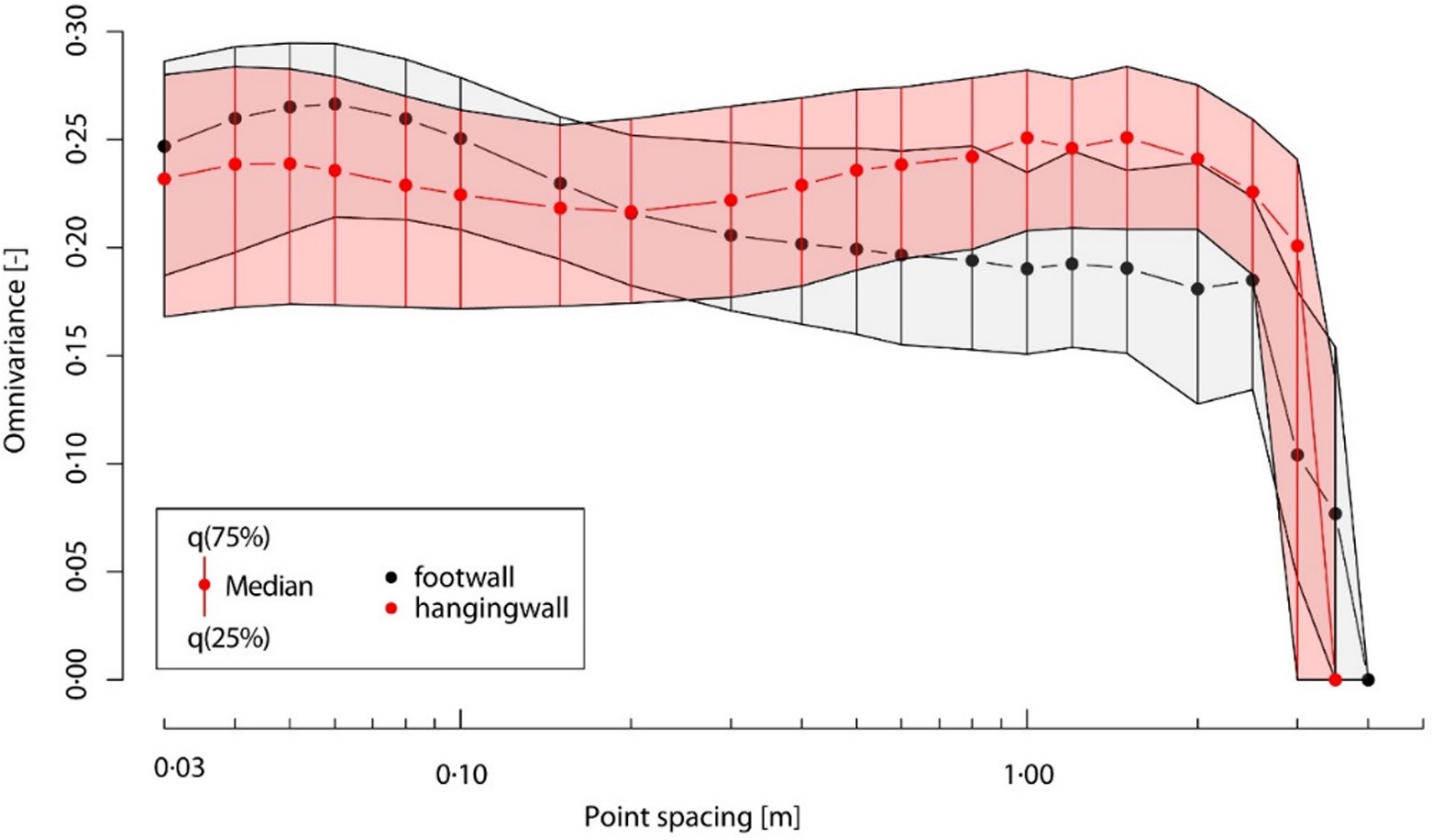
Omnivariance statistics (median and IQR) for the two investigated cave subsets calculated at varying point spacings.

**FIGURE 7:**
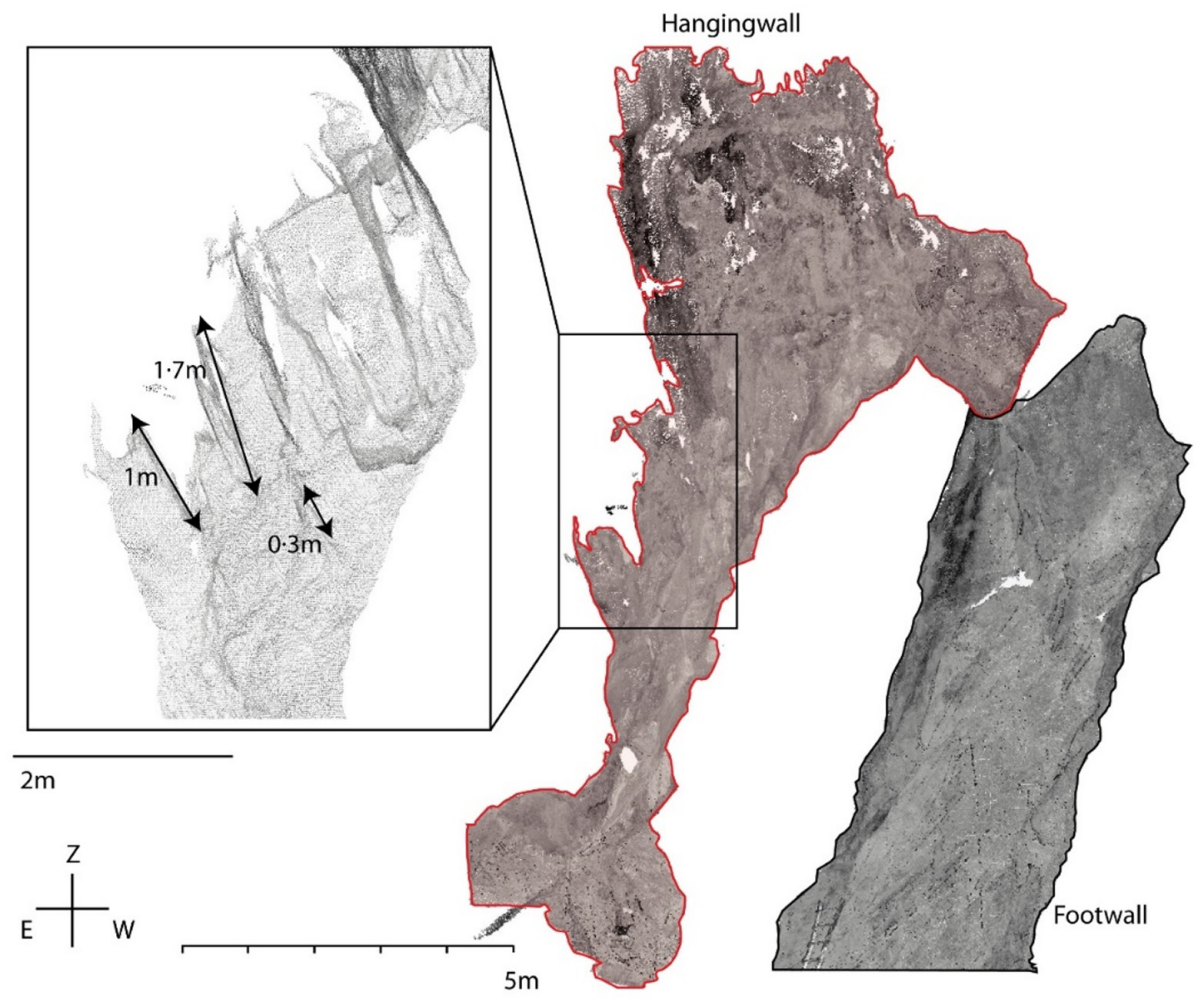
3D view of the investigated subsets (hanging wall and footwall) and exemplary measurements of dominant structural features at the hanging wall.

Maximum omnivariance values at the footwall within a 10 point‐neighbourhood arise at point clouds with a point spacing of 5 cm (Figure 6). With increasing and decreasing point spacing the omnivariance values decline. Comparably, also the hanging wall indicates a local maximum of omnivariance values at 5 cm. In contradiction to the footwall, the hanging wall additionally demonstrates a local minimum at 20 cm until the omnivariance again starts to increase with decreasing point spacing. Simultaneously the footwall specifies comparable omnivariance values at the same (20 cm) point spacing. Point spacings between 100 cm and 150 cm entail highest omnivariance values at the hanging wall subset. Hence distinct differences and similarities in the surface roughness at different cave parts and at different spatial resolutions are present and can further be used as indicators for the size of dominant surface structures. Overall, a point spacing of 20 cm indicates a spatial resolution, where the two subsets are expected to describe similar structures since no distinct differences in the respective omnivariance values are observed. Each maximum within the point‐spacing‐omnivariance comparison can be associated to a frequent occurrence of structural features engaging a comparable spatial extent. Supported by visual comparison, it becomes obvious that the hanging wall subset includes 0.3 m to 1.7 m sized features that cannot be found on the footwall (Figure 7).

## DISCUSSION

Following the presented data acquisition and registration approach, 255‐point clouds were acquired by three TLS campaigns (15 May, 23 June and 3 July 2020) in and around the ice cave resulting in a detailed 3D reconstruction. Challenging atmospheric and topographic conditions within the cave were tackled by an efficient acquisition and filtering approach. Automated registration showed success for an epoch‐wise multi‐stational alignment of overlapping point clouds containing static topographic conditions. However, minor intervention and assistance were required especially when a greater distance between the scan positions had to be introduced. This was commonly the case at very steep parts with only few possibilities for tripod assembling.

Co‐registration of multi‐temporal point clouds required the application of a classification approach ensuring the identification of dynamic areas (snow/firn/ice) within the cave. For this purpose, the combination of two classification approaches successfully separated points representing above ground objects (e.g., vegetation), ground surface, cave's surface and snow/firn/ice surfaces within the cave. Although the accurate performance of the multi‐temporal registration suggests that most of the dynamic and change‐affected parts were removed, debris‐covered ice parts are not detectable by using the presented classification approach. Their detection would require geophysical investigation methods as for example ground‐penetrating radar (e.g., Securo et al., 2022).

The resulting 3D cave map provides a unique data set which was used to study and measure cave structures, estimate the cave's internal volume, map the extent of snow/firn/ice and assess its short‐term variations. Although the interaction of a laser pulse with a snow or ice surface can cause penetration depths of up to 10 cm depending on wavelength and surface properties (e.g., Deems et al., 2013), we did not find evidence of such influences on the accuracy of the recorded laser pulse signals. Furthermore, given laser scanner's wavelength of 1550 nm the vast majority of the reflected signal returns from the top 1 cm of the snow cover (Deems et al., 2013). Back‐scattered intensities of reflected signals from snow and ice surfaces were observed to be generally low. However, no distinct data gaps induced by signal attenuation were observed.

Comparisons between 15 May and 23 June 2020 imply a mean 3D surface lowering of 6 cm in the ice‐bearing part of the cave. Observed changes can be associated with both, snow subsidence due to consolidation and/or melting. Although the origin of observed surface changes during the short observation period (about 1 month) could be associated with both, it is likely that the recently observed high impact of regional warming on ice loss is made visible by calculated differences (Wind et al., 2022). Consecutive future measurements could lead to more insights into the cave's ice mass balance (Securo et al., 2022).

The cumulative occurrence of equally sized and dominant features within the scanned data was investigated by a multiscale PCA. The results indicate a clear difference in the specificity of surface structures between the hanging wall and foot wall. Furthermore, the analysis helped to identify an efficient resolution of the data applicable to numerical airflow models (Bertozzi et al., 2019; Jarosch & Obleitner, 2022). Their future applications might help in better understanding the ice dynamics within ice‐bearing caves. In combination with consecutive TLS acquisitions and accurately monitored ice‐content dynamics, the performance of model‐based ice content variations could be evaluated.

## CONCLUSIONS

The present study demonstrates the ability of TLS to derive a consolidated and classified point cloud of 2 cm resolution by exploiting site‐adapted and novel analysis tools. Derived point clouds serve as a fundamental basis to assess geometric measures and to create precise maps of the cave's outline. The presented and highly automated registration approach demonstrates the ability of multi‐temporal TLS acquisitions for an area‐wide and precise (< 1 cm) assessment of ice dynamics in Hundsalm ice cave.

Quantitative analysis of the cave's surface by multiscale PCA and complementary visual investigations of surface structures revealed distinct differences as well as similarities between hanging and footwall subsets depending on the applied spatial resolution. At a resolution of 20 cm both subsets have comparable roughness values indicating that this point spacing is a solid basis that combines computational efficiency due to the small amount of data points and a sufficient representation of the cave's surface morphology. At the more complex hanging wall, dominant surface structures are expressed by a size larger than 30 cm and thus sustained within the sparse dataset. However, highest roughness values of the footwall occur at a spatial resolution of 5 cm. Features of the same size can not be registered by the sparse 20 cm dataset.

The highly resolved data could serve as a reference for future projects aiming on the detailed assessment of ice volume changes in Hundsalm ice cave. On the other hand, insights from a multiscale surface structure analysis identified a coarser spatial resolution of the 3D data that are well suited for atmospheric turbulence modelling approaches.

## VIDEO SUPPLEMENT

A 3D flight through the cave is animated at: https://vimeo.com/477199897

## Data Availability

Pfeiffer, Jan; Rutzinger, Martin; Spötl, Christoph (2022): 3D point cloud of the Hundsalm ice cave. PANGAEA, https://doi.org/10.1594/PANGAEA.945655
